# Predictors of Left Ventricular Outflow Tract Obstruction After Primary Interrupted Aortic Arch Repair

**DOI:** 10.1007/s00246-021-02689-9

**Published:** 2021-08-02

**Authors:** Nina A. Korsuize, Abraham van Wijk, Felix Haas, Heynric B. Grotenhuis

**Affiliations:** 1 Department of Pediatric Cardiothoracic Surgery, Wilhelmina Children’s Hospital, University Medical Center Utrecht, Utrecht, The Netherlands; 2 Department of Pediatric Cardiology, Wilhelmina Children’s Hospital, University Medical Center Utrecht, Lundlaan 6, P.O. Box 85090, 3508 AB Utrecht, The Netherlands

**Keywords:** Interrupted aortic arch, Left ventricular outflow tract obstruction, Systematic review, Echocardiography, Risk factors

## Abstract

Left ventricular outflow tract obstruction is an important complication after interrupted aortic arch repair and subsequent interventions may adversely affect survival. Identification of patients at risk for obstruction is important to facilitate clinical decision-making and monitoring during follow-up. The aim of this review is to summarize reported risk factors for left ventricular outflow tract obstruction after corrective surgery for interrupted aortic arch. A systematic search of the literature was performed across the PubMed and EMBASE databases. Studies that reported echocardiographic and/or clinical predictors for left ventricular outflow tract obstruction in infants that underwent biventricular repair of interrupted aortic arch were included. From the 44 potentially relevant studies, eight studies met the inclusion criteria. Postoperative left ventricular outflow tract obstruction requiring an intervention was common, with an incidence ranging between 14 and 38%. Manifestation of postoperative left ventricular outflow tract obstruction was associated with a smaller pre-operative size of the aortic root (sinus of Valsalva), sinotubular junction, and aortic annulus. Anatomic and surgical risk factors for left ventricular outflow tract obstruction were the presence of an aberrant right subclavian artery, use of a pulmonary homograft or polytetrafluoroethylene interposition graft for aortic arch repair, and the presence of a small- or medium-sized ventricular septal defect. In patients with a borderline left ventricular outflow tract that undergo a primary repair, these (pre-) operative predictors can provide guidance for optimal surgical decision-making and for close monitoring during follow-up of patients at increased risk for developing left ventricular outflow tract obstruction after corrective surgery.

**Supplementary Information:**

The online version contains supplementary material available at 10.1007/s00246-021-02689-9.

## Introduction

Interrupted aortic arch (IAA) is a rare congenital lesion comprising approximately 1.5% of all congenital heart disease. IAA is defined as a lack of luminal continuity between the ascending and the descending thoracic aorta [1]. The preferred treatment of IAA consists of a single-staged biventricular repair with closure of the ventricular septal defect (VSD) and reconstruction of the arch [2]. Despite excellent peri-operative survival, successful IAA repair can be complicated by left ventricular outflow tract obstruction (LVOTO), which may be present preoperatively or may (re-)occur during follow-up. Several anatomic substrates for LVOTO can be identified in IAA at a subvalvar, valvar, or supravalvar level, or as a combination of the three (multilevel stenosis). Subvalvar LVOTO is related to the posterior and leftward malalignment of the conal or outflow septum, the most commonly associated anomaly in IAA [3–5]. A prominent muscle bundle (muscle of Moulaert) on the left ventricular free wall can also contribute to LVOTO by projecting into the left ventricular outflow tract (LVOT) [6]. Valvar aortic stenosis is generally caused by incomplete or abnormal development of the aortic valve (bicuspid or unicuspid morphology, poorly defined cusps, hypoplastic aortic annulus) [7]. Supravalvar LVOTO, or supravalvar aortic stenosis, encompasses all forms of obstruction distal to the aortic valve.

Identification of patients at risk of developing LVOTO after primary repair is of great importance, as this is a common cause for reintervention in IAA patients. For patients with severely hypoplastic outflow tracts (defined as an aortic annular dimension (mm) smaller than 4 mm or smaller than the patient’s weight (kg) + 1 mm), a neonatal Ross or LVOT bypass procedure may be the best strategy [8, 9]. Patients with a sufficiently large LVOT (defined as an aortic annular dimension larger than the patient’s weight (kg) + 1.5 mm)) have low reoperation rates for LVOTO, whereas IAA patients with a borderline LVOT are at highest risk of developing LVOTO, postoperatively. Identification of pre-operative risk factors for the occurrence of LVOTO in these patients with borderline LVOT after primary repair is the aim of this review, as a set of pre-operative predictive values for the manifestation of LVOTO in these patients would be valuable in clinical practice. A number of studies have assessed echocardiographic and surgical predictors of postoperative LVOTO in patients with IAA, but to our best understanding no systematic review of these studies has been conducted. Therefore, the aim of this review was to critically appraise the available studies on the occurrence of LVOTO after primary IAA repair in order to provide clinicians with an overview of current evidence regarding predictive factors for LVOTO during follow-up. This review is also intended to identify gaps in the literature in order to provide recommendations for future research.

## Materials and Methods

### Search Strategy

A systematic search of the literature was performed across the PubMed and EMBASE databases. Additionally, the reference lists of relevant articles were screened for studies. Keywords of the search string included: ‘interrupted aortic arch,’ ‘left ventricular outflow tract obstruction,’ ‘reoperation,’ ‘reintervention,’ and ‘risk factors’. Synonyms and Medical Subject Headings (MESH) were utilized in the search. The protocol for this review has not been registered.

### Article Selection

A bibliographic software package (Mendeley) was used, and duplicates were removed. Article screening and selection were performed by the primary author, and a second researcher was consulted in case challenging decisions had to be made regarding inclusion of a study. Studies were selected based on the predetermined inclusion and exclusion criteria. Inclusion criteria were (1) main population consisting of infants who underwent biventricular repair of IAA, (2) LVOTO manifestation reported as a primary or secondary outcome measure, (3) echocardiographic or clinical predictors reported for the manifestation of LVOTO, and (4) mean follow-up time after surgery of at least 1 year. Exclusion criteria were (1) language other than English, (2) editorials, letters, conference abstracts, and expert opinions, (3) case reports with data of < 4 patients, (4) cohort with IAA patients who underwent LVOT bypass procedures or Ross procedures, and (5) cohort with single ventricle patients.

### Quality Assessment

Critical assessment of all included studies was performed using the Quality In Prognostic Studies (QUIPS) tool developed by Hayden et al. [10]. This tool guides critical appraisal of studies of prognostic factors by assessing bias in six essential domains: study participation, study attrition, prognostic factor measurement, confounding measurement, outcome measurement, and analysis and reporting. For each domain a grade of low, moderate, or high risk was assigned. Each study was evaluated by the main researcher, after which all results were discussed with a second researcher. Any differences were resolved by consensus and resulted in a single rating for each domain.

### Analysis

A meta-analysis was not feasible for this review since not only the population and methods but also the prognostic factors and outcome measures differed significantly between the available studies. Moreover, in many studies not all original data were presented or some type of selective reporting prevented full data collection. This large heterogeneity between studies is not unique for systematic reviews on prognostic studies, and therefore a narrative analysis based on statistical significance of each prognostic factor (*p*-value or odds ratio with confidence interval) was performed in this study. Data analysis and graphing were performed using Review Manager (RevMan) version 5.3 and Microsoft Excel (Microsoft Inc.). This systematic review is presented according to the PRISMA guidelines on reporting reviews [11].

## Results

### Study Selection

**Fig. 1:**
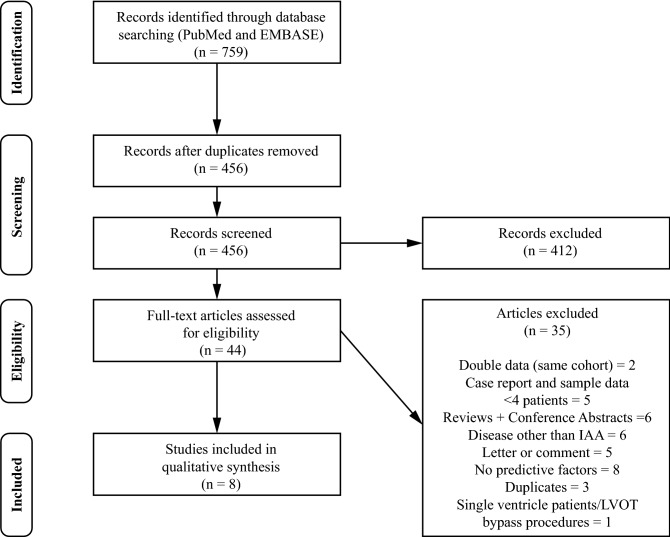
PRISMA flow chart of systematic review search

The literature search resulted in identification of 759 records. After importing in Mendeley and removal of duplicates, the title and abstract were screened for the remaining articles. The full text was acquired and read for 44 articles, of which eight were deemed relevant and subsequently included in the systematic review. Reasons for exclusion of studies are displayed in Fig. 1.

### Study Characteristics

All eight studies included in the systematic review focused on neonates who were diagnosed with IAA and had undergone primary surgical repair. All studies reported predictors for the occurrence of LVOTO after repair but differed in their definition of significant LVOTO. Three studies investigated predictive factors of LVOTO based on echocardiographic cut-off values [12, 17, 19]; the other five studies used surgical and/or catheter-based intervention for LVOTO as their outcome measure [13–16, 18]. All but two studies were single cohort studies. Geva et al. described a patient cohort from two hospitals, while the study by Jegatheeswaran et al. was based on a large population of patients from 33 different Congenital Heart Surgeons' Society (CHSS) member institutions [12, 13]. All eight studies were retrospective and had small sample sizes (ranging from 14 to 77) with the exception of the CHSS study (447 infants with IAA). All studies were conducted in the USA with the exception of the CHSS study, which was a combined effort of centers in the USA, Canada, and Brazil [13]. All study centers were either tertiary or quaternary referral hospitals. Data collection in six studies was conducted for a period of 10 years or more [13–18]. An overview of the characteristics of populations and methodological features of each study is displayed in Table 1.

**Table 1:** Demographic data from studies included in review

References (author, year)	*N*^a^	Time period	Sex (%M)^b^	IAA morphology and repair		
				IAA type	Other anomalies	Repair type
Geva (1993) [12]	37	1984–1991	51.4	6 type A, 31 type B	–^c^	–
Apfel (1998) [17]	23	1986–1997	–	3 type A, 20 type B	IAA + VSD	Primary one-stage repair
Salem (2000) [19]	14	1992–1996	64.3	4 type A, 10 type B, 2 type C	IAA + VSD + subaortic stenosis	Primary one-stage repair
Suzuki (2006) [18]	27	1991–2001	63.0	6 type A, 21 type B	IAA + VSD	Primary one-stage repair ± myectomy/myotomy
Hirata (2010) [16]	38	1994–2006	55.3	11 type A, 26 type B, 1 type C	IAA + VSD	Primary one-stage repair
Jegatheeswaran (2010) [13]	447	1987–1997	50.3	125 type A, 318 type B, 3 type C	All included	Several types of repair
Chen (2013) [14]	70	1995–2009	52.9	16 type A, 54 type B	IAA + VSD	Primary one-stage repair
Abarbanell (2018) [15]	77	2003–2013	59.7	16 type A, 60 type B, 1 type C	–	Primary repair (*n* = 60) and Yasui repair (*n* = 17)

*IAA* interrupted aortic arch, *CHSS* Congenital Heart Surgeons’ Society, *VSD* ventricular septal defect

^a^Sample size

^b^Percentage male sex

^c^Not reported

### Assessment of Quality

**Fig. 2:**
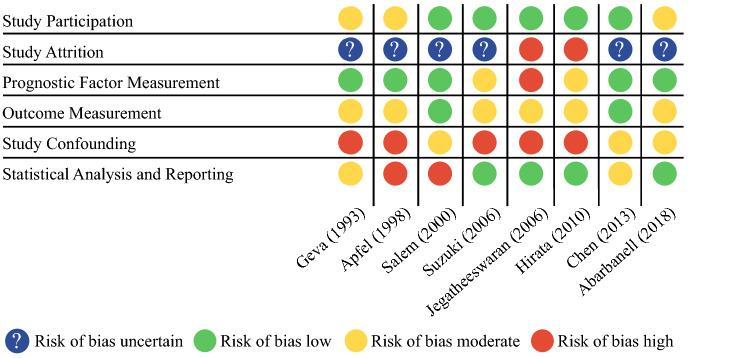
Results of quality assessment (QUIPS) of studies included in review

The results of the quality assessment of the included studies are shown in Fig. 2. In the domains study participation and outcome measurement there were no studies with a high risk of bias. On the other hand, the category study confounding was deemed of moderate or high risk for all studies involved. Study attrition was often reported as an uncertain risk of bias, as many studies did not report if patients were lost to follow-up. Overall, most studies were of moderate quality.

### Characteristics of Patients

The mean age of the patients at time of surgery was 1–1.5 weeks. The study cohort consisted of predominantly male patients (57%, range 50–64% [13, 19]). Most patients had a diagnosis of IAA type B (range 63–87%) [17, 19], and IAA type A was present in 13–29% of patients [16, 17]. Only four studies reported patients with type C (range 0.7–13%) [13, 19].

Studies differed with regards to their inclusion of patients. The majority of the studies included only patients without associated anomalies [14, 16–19]. One study included all patients with IAA and associated anomalies [13], and two studies did not report on this [12, 15].

### Analysis in Studies

The statistical method used in the majority of the studies (*n* = 5) was univariable analysis [12, 16–19]. Multivariable analysis was conducted in three studies [13–15]: the CHSS study had a large population of IAA patients for their multivariable analysis of LVOTO risk factors, while the other two studies included < 100 patients in their analysis, respectively [14, 15]. To avoid non-significant results in multivariable analysis, a minimum of ten events per variable has been proposed to maintain validity of the multivariable model, which was not met in these studies [20].

### Predictive Factors

Twenty-four predictive factors were reported to be significantly associated with the occurrence of LVOTO. Two categories can be identified: pre-operative echocardiographic parameters and surgical characteristics. The predictive factors reported to be of statistical significance by each study are presented in Table 2. Additional information on the effect sizes of all predictive factors is depicted in Online Resource 1.

**Table 2:** Predictive factors for the manifestation of left ventricular outflow tract obstruction identified by included studies

Predictive factor	Number of studies	Total number of patients	Univariable analysis Significant/tested/% significant	Multivariable analysis Significant/tested/% significant
IAA type B	4	631	2/2 (100%)	0/2 (0%)
Aberrant origin of right subclavian artery	4	631	1/2 (50%)	1/2 (50%)
Conal septum malalignment/hypoplasia	3	121	1/3 (33.3%)	
Homograft pulmonary artery used for arch repair	1	447		1/1 (100%)
Most recent procedure is index procedure	1	447		1/1 (100%)
VSD small/medium in size	1	447		1/1 (100%)
PTFE interposition graft used to repair arch	1	447		1/1 (100%)
LVOT CSA	3	121	3/3 (100%)	0/2 (0%)
Indexed LVOT CSA	3	107	1/3 (33.3%)	
LVOT diameter	3	111	2/3 (66.6%)	0/2 (0%)
Indexed LVOT diameter	4	101	1/4 (25%)	
LVOT *z*-score	1	23	1/1 (100%)	
LVOT CSA/AoV CSA	2	107	1/2 (50%)	
LVOT CSA/VSD CSA	2	107	1/2 (50%)	
AoV diameter	3	107	(1/2) (50%)	1/2 (50%)
Indexed AoV diameter	3	74	1/3 (33.3%)	0/1 (0%)
AoV *z*-score	3	64	1/3 (33.3%)	1/1 (100%)
AoV CSA/PV CSA	2	107	1/2 (50%)	
AoV diameter/PV diameter	3	74	1/3 (33.3%)	0/1 (0%)
AoV diameter in relation to weight in kg	1	38	1/1 (100%)	
Aortic root diameter	2	147	1/1 (100%)	2/2 (100%)
STJ diameter	2	147	0/1 (0%)	1/1 (100%)
AAo diameter	2	84	1/2 (50%)	0/1 (0%)
Indexed AAo diameter	2	51	1/2 (50%)	0/1 (0%)

*AAo* ascending aorta, *AoV* aortic valve, *CSA* cross-sectional area, *DAo* descending aorta, *IAA* interrupted aortic arch, *LVOT* left ventricular outflow tract, *LVOTO* left ventricular outflow tract obstruction, *PTFE* polytetrafluoroethylene, *PV* pulmonary valve, *STJ* sinotubular junction, *VSD* ventricular septal defect

#### Pre-operative Echocardiographic Indices

##### Anatomic Findings

An aberrant origin of the right subclavian artery (RSCA) appeared to be a risk factor for subsequent manifestation of LVOTO: 50% in univariable and multivariable testing. This factor was tested in the largest population in this review (4 studies, *n* = 631) [12–15]. Posterior malalignment or hypoplasia of the conal septum appeared to be of little predictive value for postoperative LVOTO, as only one out of three studies reported a statistically significant association in univariable analyses (3 studies, *n* = 121) [12, 14, 19]. VSD type was part of univariable analysis in only one study and did not prove to be associated with a higher risk for LVOTO (*n* = 37) [12]. The presence of a bicuspid aortic valve was not predictive of LVOTO in univariable analysis (1 study, *n* = 70) [14] nor was it in a multivariable model (1 study, *n* = 77) [15].

IAA type B was reported as a risk factor in 100% of univariable analyses (2 studies, *n* = 107) [12, 14]; however, multivariable analyses did not show any significance (2 studies, *n* = 524) [13, 15].

##### Subvalvar Measurements

Cross-sectional area and diameter of the LVOT were predictive of an increased risk of LVOTO in univariable testing (100% and 66.7%, respectively). However, this did not prove significant in the multivariable models (4 studies, *n* = 148) [12, 14, 18, 19]. Indexed LVOT cross-sectional area and diameter were moderately predictive in univariable analyses (33.3% and 25%, respectively), although these parameters were not tested in multivariable models (5 studies, *n* = 171) [12, 14, 17–19]. Geva et al. reported that a cross-sectional area smaller than 0.7 cm^2^/m^2^ is a sensitive predictor of LVOTO at a later stage. Apfel et al. reported a cut-off value of 1.6 cm^2^/m^2^ [12, 17]. Only one study reported on the LVOT *z*-score as a risk factor, which came out significant in univariable testing: IAA patients who developed postoperative LVOTO had a mean pre-operative LVOT *z*-score of − 7.6 compared to − 6.4 in the patients that did not develop LVOTO (*n* = 23) [17]. Increased Doppler velocities across the LVOT at pre-operative assessment were not predictive of subsequent LVOTO manifestation (2 studies, *n* = 51) [12, 19].

##### Valvar Measurements

Aortic valve diameter was predictive of subsequent LVOTO in 50% of univariable testing (2 studies, *n* = 84) [14, 19], but not in multivariable testing (1 study, *n* = 77) [15]. Aortic valve *z*-score was predictive of a higher risk for LVOTO occurrence in 33.3% of univariable testing (3 studies, *n* = 64) [17–19] and in 100% of multivariable testing (1 study, *n* = 14) [19]. Salem et al. reported a pre-operative aortic valve diameter smaller than 4.5 mm as a risk factor for postoperative LVOTO, corresponding to a *z*-score smaller than − 5 [19]. The study by Hirata et al. identified a pre-operative aortic annulus size less than the patient’s weight (kg) + 1.5 mm as a predictor for the need of a LVOTO reoperation [16]. Aortic valve cross-sectional area and indexed aortic valve cross-sectional area were not predictive of an increased risk of LVOTO in univariable analyses (2 studies, *n* = 107) [12, 14].

##### Supravalvar Measurements

Smaller aortic root size (diameter at sinus of Valsalva level) at pre-operative evaluation was predictive of LVOTO occurrence in 100% of the univariable testing and 100% of the multivariable models in two recent studies that tested for this variable (*n* = 147) [14, 15]. In the study by Abarbanell et al. sinotubular junction (STJ) size was a risk factor for LVOTO in their multivariable model, whereas STJ size did not meet statistical significance in the univariable analysis by Chen et al. [14, 15]. The study by Chen et al. reported an aortic root size smaller than 6.5 mm as a risk factor for LVOTO reinterventions. Abarbanell et al. reported an aortic root *z*-score less than − 2.5 as a risk factor for reoperation for LVOTO. Ascending aorta diameter and indexed ascending aorta diameter were not associated with an increased risk of LVOTO in the three studies that investigated this factor, with only a significant result in 50% of univariable testing and 0% in multivariable testing (3 studies, *n* = 121) [12, 14, 19]. Indexed descending aorta diameter was reported by one study and did not come out significant in univariable testing (*n* = 37) [12].

##### Echocardiographic Ratios

Several echocardiographic ratios were tested for their potential to predict LVOTO occurrence; however none proved to be of predictive value. The aortic valve-to-pulmonary valve diameter ratio was tested most often, being significant in 33.3% of univariable analyses (3 studies, *n* = 74) [12, 17, 19], but not in multivariable analysis (1 study, *n* = 14) [19]. The aortic valve-to-descending aorta and LVOT-to-descending aorta ratios were assessed in univariable testing in one study (*n* = 23), which was not significant either [17]. The LVOT cross-sectional area-to-aortic valve cross-sectional area and the LVOT cross-sectional area-to-VSD cross-sectional area ratios were significant in 50% of univariable analyses (2 studies, *n* = 107) [12, 14]. The ascending aorta-to-descending aorta diameter ratio was included in one univariable analysis and was found not to be significant (*n* = 14) [19].

#### Patient and Surgical Characteristics

Two studies included patient and surgical characteristics into their multivariable analyses. Jegatheeswaran et al. reported that the use of a pulmonary homograft for aortic arch repair was associated with an early risk of reintervention for LVOTO [13]. The use of a prosthetic polytetrafluoroethylene (PTFE) interposition graft and the presence of a small- or medium-sized VSD were risk factors for late LVOT reintervention after primary IAA repair. [13]. The presence of 22q11 deletion syndrome was not associated with an increased risk for the manifestation of LVOTO, neither was a subaortic stenosis intervention at initial repair [14].

##### Development of LVOTO

**Fig. 3:**
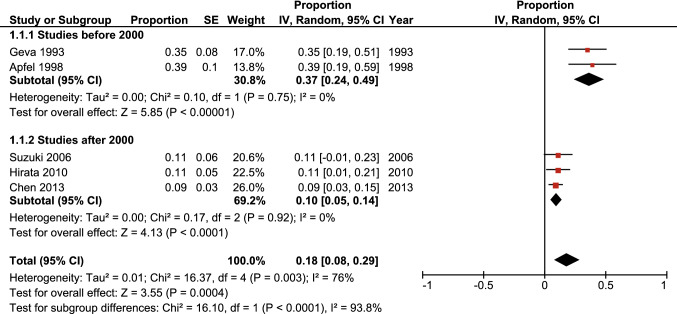
Left ventricular outflow tract obstruction development in the first year after initial interrupted aortic arch repair as reported by included studies

**Fig. 4:**
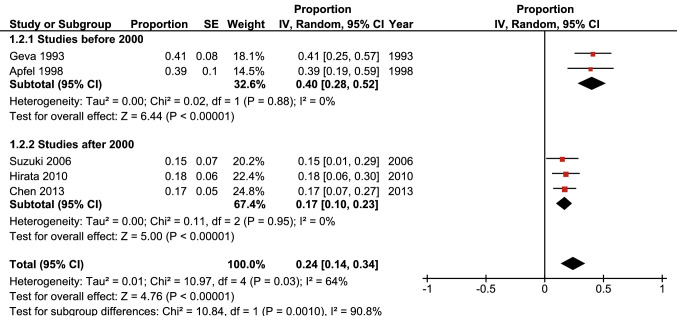
Left ventricular outflow tract obstruction development in the first 5 years after initial interrupted aortic arch repair as reported by included studies

Predictive factors for the development of LVOTO are best discussed within the time frame in which have a predictive effect. For the completeness of this review an analysis was performed to depict the time frame of LVOTO development in the included studies. Five out of the eight studies reported sufficient data for this analysis, both for the 1-year and 5-year time periods (see Figs. 3 and 4).

It should be noted that these forest plots have been created based on data from retrospective cohorts and should therefore not be interpreted as the total number of LVOTO patients, as after the study period ended the patients were still at risk. The plots show the proportion of patients that developed LVOTO in the first year after surgery and in the first 5 years after surgery. It is apparent that the proportion of patients that develops LVOTO is highest within the first year after initial IAA repair.

## Discussion

The aim of this systematic review was to identify (pre-) operative predictors for manifestation of LVOTO after primary IAA repair. Eight relevant articles were suitable for analysis. We found the following:

1. Postoperative LVOTO is a frequent complication after IAA repair (incidence range 3.6–67%), often requiring intervention during follow-up (13.5–38%).
2. Pre-operative echocardiographic risk factors for LVOTO after IAA repair were size of aortic annulus (< weight (kg) + 1.5 mm), sinotubular junction, and aortic root (< 6.5 mm).
3. Other risk factors for LVOTO were the presence of an aberrant RSCA, use of a pulmonary homograft or PTFE interposition graft for aortic arch repair, and the presence of a small- or medium-sized VSD.

The incidence of LVOTO after IAA repair reported in literature demonstrates a wide range between 3.6 and 67% [21]. The range of incidences may be explained by the different measurement strategies and cut-off values for LVOTO, as well as different follow-up times and surgical strategies between the studies (13 and 20). Because of this heterogeneity, we herein report the number of patients requiring reintervention for LVOTO. In our included studies, the incidence for LVOT intervention ranged between 13.5 and 38%, which is in line with the 25% 5-year risk as generally reported in literature [21]. Studies included in this review reported that the risk of LVOTO occurrence is highest in the first year after primary IAA repair: Apfel et al. reported that all cases of LVOTO in their study were diagnosed in the first year after primary repair [17], Salem et al. reported that LVOTO was diagnosed at a mean of 9.4 months after initial surgery [19], and Geva et al. reported that 87% of all LVOTO patients in their cohort were diagnosed within the first month after primary surgery [12]. The nature of LVOTO preoperatively differs from postoperative LVOTO. Whereas pre-operative LVOTO typically consists of posterior malalignment of the conal septum, a combination of a hypoplastic aortic annulus and the presence of a circumferential subaortic membrane, is typically responsible for postoperative LVOTO. The latter pathology develops after the immediate postoperative period and may be secondary to turbulent flow dynamics within the LVOT [14]. Interestingly, a VSD patch or conal septal protrusion into the LVOT did not predict subsequent LVOTO [14].

Several pre-operative echocardiographic indices could be identified as predictors for subsequent LVOTO. Smaller aortic root size on pre-operative echocardiography was identified in multivariable regression analysis as an independent predictor of reintervention for LVOTO [14]. Logistic regression modeling showed that neonates with an aortic root size of less than 6.5 mm were at greater risk for reintervention when compared to those with aortic root sizes larger than 6.5 mm, with a reintervention rate of 44% and 12%, respectively [14]. The corresponding odds ratio of 9.9 supports this finding. The study by Abarbanell et al. reported similar findings, as decreasing aortic root *z*-score more than doubled the odds of LVOTO intervention (odds ratio 2.7), with the inflection point for this risk at a *z*-score of − 2.5 [15]. Smaller sinotubular junction size was also reported as an independent risk factor for LVOTO intervention following primary repair [15]. Salem et al. reported in their study that the aortic valve diameter was the most sensitive predictor of subsequent LVOTO manifestation, which was present in all patients with a pre-operative aortic annulus diameter of 4.5 mm or less (corresponding to a *z*-score of − 5 or less) [19]. Hirata et al. reported that the pre-operative measurement of the difference between the aortic annulus size and the patient’s weight was a good predictor for subsequent LVOTO reintervention [16]. If the aortic annulus was larger than the patient’s weight + 1.5 mm (“large annulus”), excellent survival without further reoperation was predicted. When the aortic annulus size was below the patient’s weight + 1.5 mm, reoperations were required more frequently [16]. More specifically, a LVOT bypass procedure was recommended for patients with aortic annulus size smaller than weight + 1.0 mm [16]. Chen et al. however reported that although there was a trend toward smaller aortic annulus sizes in the LVOT reintervention group in their study, this was not statistically significant. Several patients in their cohort with an aortic annulus diameter of 3.5–4.0 mm did not require reintervention during follow-up [14].

Doppler velocities across the LVOT at pre-operative assessment were not predictive of an increased risk of LVOTO in the studies included in this review [12, 19]. This may be due to the fact that typically a large VSD is present in IAA, which decompresses the left ventricle and thus minimizes the pressure gradient across the LVOT. In contrast to what may be expected, LVOT diameter and cross-sectional area did not prove to be of significant predictive value in multivariable models in the studies included in this review. As discussed by Hirata et al. this can be attributed to the fact that measurement of the subaortic region is relatively inconsistent, as these measurements depend on the patient’s volume status and the dynamic phase of systole and diastole [16].

Several anatomic and surgical indices were also identified as predictive for subsequent LVOTO. The presence of an aberrant RSCA was found to be predictive for postoperative LVOTO in half of the studies that tested for this variable [12–15]. An aberrant RSCA is most likely an indirect predictor of postoperative LVOTO manifestation: antegrade flow across the LVOT will be reduced in utero, as the ascending aortic flow only supplies the right and left carotid arteries. Hence, it is plausible that the reduced flow across the LVOT in utero results in more pronounced hypoplasia of the LVOT. Since the presence of an aberrant RSCA was not associated with a higher risk of postoperative LVOTO manifestation in all studies, this can be the result of adjustment for the interaction in their statistical model [14, 15].

The study by Jegatheeswaran et al. found that the presence of a small- or medium-sized VSD and the use of a pulmonary homograft or PTFE interposition graft for repair of the arch were risk factors for subsequent LVOTO [13]. Patch augmentation may have been necessary for the repair of hypoplastic aortic arches. The need for more extensive arch reconstruction with patch augmentation is possibly associated with a smaller LVOT, which is inherently associated with LVOTO. It was also reported that most of the patients who required an intervention for LVOTO underwent the initial IAA repair as their most recent procedure. The latter was reportedly due to the large number of patients with small LVOT sizes that were not addressed at initial IAA repair, although the study by Jegatheeswaran et al. reported no significant trend for the acute risk of LVOT procedures and an increase in the chronic risk after each subsequent LVOT procedure. Other studies included in this review reported a higher incidence of LVOT procedures in the early phase after initial IAA repair, with almost no late interventions [12, 17, 19]. In the study by Jegatheeswaran et al. no clear rationale was provided as to why the presence of a small- or medium-sized VSD would lead to a higher risk of LVOTO manifestation [13]. It can be hypothesized however that the presence of a large VSD results in an underestimation of the aortic annulus size on echocardiographic evaluation, as there will be an increased left-to-right shunt.

## Limitations

This review has several limitations; first, no meta-analysis could be performed. After consultation with our institutional statistical support center, it was decided that conducting a meta-analysis was not feasible because of the large heterogeneity between studies. All studies were retrospective in nature, so there is a higher likelihood that potential risk factors are missing, as data were not available (or known for only part of the sample) for these factors in the study cohorts. Especially factors that are not routinely measured in a clinical setting may be affected. Unfortunately, only one of the studies reported on *z*-scores for pre-operative echocardiographic predictors, even though *z*-scores are more robust in the clinical setting of small children when compared to absolute values.

The large variability between the studies was another limitation for this study. The majority of potential risk factors was only measured in one or two studies. Similarly, the measurement methods used for the echocardiographic risk factors differed slightly between studies, which also limits comparability. Furthermore, data collection in six studies was conducted for a period of 10 years or more. Although long-term follow-up can be beneficial to include enough patients with IAA for statistical purposes, it may also lead to era bias since hospital policies, medication, and (surgical) treatments can be expected to have changed over time, affecting patient outcomes such as mortality and degree of LVOTO. In keeping with this, some studies included in this review started inclusion as early as the 1980s, whereas others focused on populations up until 2013, which makes comparison challenging. The statistical method used in most studies was univariable analysis followed by multivariable analysis of the identified risk factors. Limitations also apply to risk factors that reached level of significance in univariable analysis but not in multivariable analysis. Many studies have included more variables in their models than warranted by the number of patients included, which also should be taken into consideration while interpreting the results. The small sample sizes of the studies also limit the generalizability of the results (with the exception of the study by Jegatheeswaran et al.). Lastly, significant variation in LVOTO endpoints was present between studies.

## Conclusion

The identification and measurement of risk factors for the manifestation of LVOTO in patients after IAA repair have the potential to improve clinical and surgical decision-making. This review identified several predictive factors that were assessed in eight eligible studies. The factors with highest predictive value were a smaller pre-operative size of the aortic root (sinus) (< 6.5 mm or *z*-score < − 2.5), smaller STJ size, an aortic annulus size < weight (kg) + 1.5 mm, the presence of an aberrant RSCA, the use of a pulmonary homograft or PTFE interposition graft for aortic arch repair, and the pre-operative presence of a small- or medium-sized VSD.

Careful evaluation of these (pre-) operative predictors can be helpful for surgical planning in patients with IAA. In patients with a borderline LVOT that undergo a primary repair, the pre-operative predictors can provide guidance as to which patient is at higher risk for developing LVOTO and therefore should be monitored more closely during follow-up. The latter may lead to better surgical decision-making in the future. Importantly, significant heterogeneity of study designs, small sample sizes, and potential confounding factors pose difficulty for interpretation of the study results and emphasize the need for more (prospective) research in this field.

## Supplementary Information

Below is the link to the electronic supplementary material.

Supplementary file1 (XLSX 12 kb)

## Data Availability

Not applicable.

## References

[1] Backer CL, Mavroudis C (2000). Congenital Heart Surgery Nomenclature and Database Project: patent ductus arteriosus, coarctation of the aorta, interrupted aortic arch. Ann Thorac Surg.

[2] Kaza AK, Thiagarajan RR (2016). Left ventricular outflow tract obstruction: coarctation of the aorta, interrupted aortic arch, and borderline left ventricle. Pediatr Crit Care Med.

[3] Celoria GC, Patton RB (1977). Congenital absence of the aortic arch. Am Heart J.

[4] Freedom RM, Bain HH, Esplugas E, Dische R, Rowe RD (1977). Ventricular septal defect in interruption of aortic arch. Am J Cardiol.

[5] Van Praagh R, Bernhard WF, Rosenthal A, Parisi LF, Fyler DC (1971). Interrupted aortic arch: surgical treatment. Am J Cardiol.

[6] Fiore AC, Peterson RE, Huddleston CB, Ungerleider RM, Meliones JN, Nelson McMillan K, Cooper DS, Jacobs JP (2019). Repair of interrupted aortic arch with ventricular septal defect. Critical heart disease in infants and children.

[7] Hraska V, Murin P, Hraška V, Murín P (2012). Left ventricular outflow tract anomalies. Surgical management of congenital heart disease I: complex transposition of great arteries, right and left ventricular outflow tract obstruction, Ebstein’s anomaly—a video manual.

[8] Riggs KW, Tweddell JS (2019). How small is too small? Decision-making and management of the small aortic root in the setting of interrupted aortic arch. Semin Thorac Cardiovasc Surg Pediatr Card Surg Annu.

[9] Hraška V, Murín P, Hraška V, Murín P (2015). Interruption of the Aortic Arch. Surgical management of congenital heart disease II: single ventricle and hypoplastic left heart syndrome, aortic arch anomalies, septal defects and anomalies in pulmonary venous return, anomalies of thoracic arteries and veins—a video manual.

[10] Hayden JA, van der Windt DA, Cartwright JL, Côté P, Bombardier C (2013). Assessing bias in studies of prognostic factors. Ann Intern Med.

[11] Moher D, Liberati A, Tetzlaff J, Altman DG, PRISMA group (2009). Preferred reporting items for systematic reviews and meta-analyses: the PRISMA statement. PLoS Med.

[12] Geva T, Hornberger LK, Sanders SP, Jonas RA, Ott DA, Colan SD (1993). Echocardiographic predictors of left ventricular outflow tract obstruction after repair of interrupted aortic arch. J Am Coll Cardiol.

[13] Jegatheeswaran A, McCrindle BW, Blackstone EH (2010). Persistent risk of subsequent procedures and mortality in patients after interrupted aortic arch repair: a Congenital Heart Surgeons’ Society study. J Thorac Cardiovasc Surg.

[14] Chen PC, Cubberley AT, Reyes K (2013). Predictors of reintervention after repair of interrupted aortic arch with ventricular septal defect. Ann Thorac Surg.

[15] Abarbanell G, Border WL, Schlosser B, Morrow G, Kelleman M, Sachdeva R (2018). Preoperative echocardiographic measures in interrupted aortic arch: which ones best predict surgical approach and outcome?. Congenit Heart Dis.

[16] Hirata Y, Quaegebeur JM, Mosca RS, Takayama H, Chen JM (2010). Impact of aortic annular size on rate of reoperation for left ventricular outflow tract obstruction after repair of interrupted aortic arch and ventricular septal defect. Ann Thorac Surg.

[17] Apfel HD, Levenbraun J, Quaegebeur JM, Allan LD (1998). Usefulness of preoperative echocardiography in predicting left ventricular outflow obstruction after primary repair of interrupted aortic arch with ventricular septal defect. Am J Cardiol.

[18] Suzuki T, Ohye RG, Devaney EJ (2006). Selective management of the left ventricular outflow tract for repair of interrupted aortic arch with ventricular septal defect: management of left ventricular outflow tract obstruction. J Thorac Cardiovasc Surg.

[19] Salem MM, Starnes VA, Wells WJ (2000). Predictors of left ventricular outflow obstruction following single-stage repair of interrupted aortic arch and ventricular septal defect. Am J Cardiol.

[20] Harrell FE, Lee KL, Matchar DB, Reichert TA (1985). Regression models for prognostic prediction: advantages, problems, and suggested solutions. Cancer Treat Rep.

[21] Mishra PK (2009). Management strategies for interrupted aortic arch with associated anomalies. Eur J Cardiothorac Surg.

